# New opportunities for kinase drug repurposing and target discovery

**DOI:** 10.1038/s41416-018-0045-6

**Published:** 2018-03-16

**Authors:** Stefan Knapp

**Affiliations:** 1 Institute for Pharmaceutical Chemistry, Johann Wolfgang Goethe-University, Max-von-Laue-Str. 9, Frankfurt, D-60438 Germany; 2 Structural Genomics Consortium, Buchmann Institute for Molecular Life Sciences, Johann Wolfgang Goethe-University, Max-von-Laue-Str. 15, Frankfurt, D-60438 Germany; 3 Structural Genomics Consortium, Nuffield Department of Clinical Medicine, University of Oxford, Oxford, OX3 7DQ UK; 4 German Cancer Network (DKTK), Frankfurt/Mainz site, Frankfurt, D-60438 Germany

## Abstract

Protein kinases are major drug targets for oncology. The large size of the kinome, active site conservation and the influence of activation states on drug binding complicates the analysis of their cellular mode of action. In a recent article in *Science*, Klaeger et al. analysed cellular targets of 243 drug candidates providing a large repository of data for drug repurposing.

Deregulation of signalling pathways is a hallmark of cancer. It is therefore no surprise that protein kinases have emerged as one of the most successful target classes for the development of cancer treatments. Currently 37 small molecule kinase inhibitors are approved by the FDA; almost all of them for applications in oncology. Based on the large number of kinase inhibitors that are in clinical trials at present it is highly likely that we will see many more approvals soon. This prediction is supported by the large number of FDA and EMA approvals during the past 4 years that have contributed half of our clinical arsenal of kinase-targeted drugs^1^. Also in 2017, four additional kinase drugs have been approved by the FDA (http://www.brimr.org/PKI/PKIs.htm)^2^.

The mode of action of many kinase inhibitors is, however, difficult to understand. Although most kinase inhibitors have been developed against a specific kinase target, many are promiscuous, often inhibiting multiple kinases in key signalling pathways, but how efficiently these pathways are inhibited on a cellular level is an unresolved question. Polypharmacology may be required for treatment efficacy in diverse cancer types that display a complex landscape of genetic lesions, but the lack of understanding of the mechanisms that result in treatment benefits limits further clinical development and the identification of new disease-modulating kinase targets. The characterisation of kinase drug selectivity is an important prerequisite for our understanding of the cellular mechanisms that lead to drug response. In a recent issue of *Science*, Klaeger et al.^3^ analysed the cellular targets of 243 clinically studied kinase drug candidates using chemical proteomics. This comprehensive study provides a large repository of data for drug repurposing, target identification, and the rational design of novel kinase inhibitors. Surprisingly, a number of non-kinase off-targets have also been identified that may lead to side effects in the clinic.

Kinome-wide in vitro screening technologies have previously revealed the complexity of the target space inhibited by commonly used kinase tool compounds, as well as inhibitors that were studied clinically during that time^4–7^. The data from these profiling studies have had a major impact on kinase drug discovery by highlighting off-target liabilities and unexpected activity on kinase targets that led to repurposing, in some cases, and casted doubt on validation studies that used these promiscuous inhibitors as tool compounds. However, these selectivity profiles were generated using purified isolated catalytic domains, neglecting the effect of regulatory domains in the native full-length kinase, posttranslational modifications, and the influence of the cellular environment that may modulate inhibitor efficacy by cofactor competition, the binding of protein interaction partners, and cellular location.

Assays have now been developed to allow broad cellular profiling. First, Vasta et al.^8^ used an array of fluorescent-based assays and highlighted the importance of the cellular environment, such as cellular location, ATP, or metabolite concentration and the use of full-length kinases, but this approach used ectopically expressed kinases and cannot currently validate selectivity on a kinome-wide scale. In contrast, the kinobeads technology used in the Klaeger^3^ study makes use of cellular extracts containing endogenous full-length proteins in complex with regulatory proteins and harbouring posttranslational modifications, and considers the presence of metabolites and cofactors^9^. This assay is run in the format of a competition binding assay, where a number of promiscuous kinase inhibitors are immobilised on a bead to capture the cellular kinases. In the inhibitor-treated lysates, the kinase inhibitor will compete to bind the kinase; binding can be quantified in pulldown assays with the help of mass spectrometry. Measuring this competition in an inhibitor-concentration-dependent way, thousands of proteins can be assayed in parallel resulting in dissociation constants (*K*_D_) for every drug interaction with cellular proteins. However, the assay may miss interactions with kinases that are absent or weakly expressed in the cell lines used, have intrinsic and inhibitor independent affinity for the beads, or that are not captured by the immobilised inhibitors. Despite these limitations, validation studies showed that kinobeads capture a comprehensive set of kinases and the use of several diverse cell lines mitigates the risk that an off-target is not expressed in a certain cell type^10, 9^.

The kinobead assay has the further advantage that some non-kinase proteins can also be identified as targets of clinical kinase inhibitors. A number of non-kinase targets have been described to be potently inhibited, including proteins that are structurally very distinct from kinases, such as G-protein-coupled receptors (GPCRs)^7^ and bromodomains^11^, as well as other nucleotide-binding proteins. The non-protein kinase targets of clinical inhibitors identified by the Klaeger et al.^3^ study comprise metabolic kinases, such as pyridoxal kinase and other nucleotide binders, proteins that bind flavin adenine dinucleotide, and the haem-binding enzyme ferrochelatase. GPCRs and bromodomains cannot be detected using kinobeads as the immobilised inhibitors do not bind any of these targets. Using four diverse cell lines (K-562, MV-4-11, SK-N-BE(2), and COLO 205) a total of 253 kinases were identified binding to kinobeads. This assay format revealed that the chosen clinical kinase inhibitor set targeted 220 kinases with high affinity (in the nanomolar *K*_D_) providing an interaction map of the currently druggable kinome with clinical inhibitors.

The selectivity of clinical inhibitors ranged from largely promiscuous compounds targeting more than 100 kinases to inhibitors with exquisite selectivity for just one target, suggesting that clinical efficacy may not always require simultaneous inhibition of several kinases. Examples of highly selective compounds were the MET receptor tyrosine kinase inhibitor capmatinib, the epidermal growth factor receptor (EGFR) inhibitor lapatinib, and the checkpoint kinase 1 inhibitor rabusertib. Irreversible inhibitors are often thought to be more selective due to bond formation with cysteine residues that are only present in a few kinases^12^. The recent approval of several irreversible inhibitors targeting EGFR and Bruton’s tyrosine kinase spurred interest in these types of inhibitors; however, Klaeger et al.’s^3^ kinome bead assays showed that the covalent binding mode alone is not sufficient to confer high selectivity for the intended target.

The authors provide several examples how this large array of profiling data can be used for translational research. An obvious way is the repurposing of approved drugs. The MET/VEGFR inhibitor cabozantinib is currently approved for medullary thyroid cancer and advanced renal cell carcinoma^13^. The study by Klaeger et al.^3^ showed that cabozantinib is also a potent inhibitor of the tyrosine kinase fusion product FLT3-ITD, suggesting potential application in FLT3-ITD positive acute myeloid leukaemia (AML). Indeed, cell lines bearing the FLT3-ITD rearrangement but not wild type AML cell lines were sensitive to cabozantinib treatment, which potently inhibited phosphorylation of the FLT3 downstream target STAT5 and showed efficacy in a xenograft model. The data will be available in a database and it is likely that, based on the provided selectivity profiles, more repurposing opportunities will emerge.

Apart from drug repurposing, the data from this assay can also be used for the identification of novel kinase targets. For example, expression profiling using kinobeads and 15 non-small cell lung cancer (NSCLC) tumours identified the known cancer targets EGFR and MAP2K1 (MEK1), as well as the kinases DDR1 (discoidin domain receptor tyrosine kinase 1) and MELK (maternal embryonic leucine zipper kinase). Tissue arrays of a cohort of 375 NSCLC, squamous cell carcinoma, and adenocarcinoma patients showed no significant correlation with EGFR and DDR1 expression in a retrospective survival analysis, but these data revealed a moderate overall correlation with MELK expression levels. Separate analysis suggested poor survival of patients with high MELK levels in squamous cell carcinoma but not adenocarcinoma patients, in support of earlier studies^14^. The only clinical kinase inhibitor developed for MELK (OTS-167) had a very broad kinase activity making it not suitable as an inhibitor to study MELK function in squamous cell carcinoma, but kinobead profiling identified an additional 16 inhibitors, including the approved drug nintedanib that may be more suitable for mechanistic studies, or that may serve as a starting point for the development of selective MELK inhibitors using a pharmacological validated inhibitor scaffold.

The study by Klaeger et al.^3^ provides an exciting data source describing the target landscape of current kinase inhibitors. This large repository of selectivity data will be highly informative for basic research scientists, as well as drug discovery and clinical repurposing of approved kinase drugs. Our repository of kinase drugs and drug candidates is growing rapidly, and it is therefore highly likely that comprehensive profiling data as presented in this study will be the one of the main sources for future target identification and the development of new therapeutic concepts.
